# Marginal bone loss around cement and screw-retained fixed implant prosthesis

**DOI:** 10.4317/jced.55194

**Published:** 2018-10-01

**Authors:** Muhammad-Hasan Hameed, Farhan-Raza Khan, Robia Ghafoor, Syed-Iqbal Azam

**Affiliations:** 1 Resident, Department of Dentistry, Aga Khan University Hospital, Karachi, Pakistan; 2 Associate Professor, Department of Dentistry, Aga Khan University Hospital, Karachi, Pakistan; 3 Assistant Professor, Department of Community Health Sciences, Aga Khan University Hospital, Karachi, Pakistan

## Abstract

**Background:**

Implant-supported fixed restorations are considered as the standard treatment for replacement of missing teeth. These can be either screw or cement retained. The success or failure of implant restorations depend upon amount of marginal bone loss (MBL). The present study is to determine the MBL around cement and screw-retained implant prosthesis and to determine various predictors of the MBL.

**Material and Methods:**

A retrospective charts review was conducted at the dental clinics, Aga Khan University Hospital, Karachi from February 2017 to June 2017 in which 104 implants restorations were assessed using periapical radiographs. MBL was calculated at baseline and at 12 months and the difference was recorded on a proforma. SPSS version 21.0 was used for statistical analysis. Descriptive statistics was computed. Generalized estimation equation analysis (GEE) was applied to determine the predictors of MBL. Level of significance was kept at ≤ 0.05.

**Results:**

There were 104 implant restorations belonging to 41 patients. Screw retained prosthesis showed significantly greater MBL than cement retained prosthesis (*p*-value =0 .018) (irrespective of crowns or fixed partial dentures). Other factors that turned out to be significant predictors of MBL were male gender (*p*-value= <0.01), age >65 yrs. (*p*-value=0.028) and sites where bone grafting was performed (*p*-value=0.003).

**Conclusions:**

Male patients of age >65 yrs. with sites needing bone grafts who were provided with screw retained prosthesis (irrespective of crown or fixed partial dentures) had significantly greater marginal bone loss around implants.

** Key words:**Dental implants, dental prosthesis, implant supported dental prosthesis, alveolar bone loss.

## Introduction

Fixed implant-supported restorations have shown predictable success as a modality of treatment for missing teeth (1). These are now considered as the standard of care in preventing the adverse physical and cosmetic outcomes associated with missing teeth (2). Appearance and mastication of partially or totally edentulous patients have been greatly improved using implant based restorations (3,4). These restorations can be screw or cement retained to the implant, or both (4,5). Clinician’s own preference mainly influences the selection of retention system. Generally, the mode of retention is decided at the planning stage of the case when the merits and demerits of each system are taken into account based on the planned treatment (6).

Usually screw-retained systems are selected when there are multiple abutments present as this retention mechanism allows the removal of prosthesis for hygiene maintenance and potential repairs (7). Moreover, there is minimal marginal discrepancy at the crown implant interface when compared with the cement-retained prosthesis. However, screw loosening is a common problem with method. Moreover, esthetic considerations become vital when the implants are not installed in the desirable position (8). Cement-retained prosthesis are considered ideal where esthetics is the primary consideration. Additionally they can compensate for implants that have been placed in unfavorable angulation, to correct relationship of crown with the implant; they are easy to fabricate with lessened possible laboratory complications (9). They are more commonly used in patients requiring single crowns, because in vitro studies have proved that they exert minimum stress on the bone tissue and implant components than screw-retained prostheses (4).

To evaluate the success or failure of implant, changes in the marginal bone level and osseo-integration are the chief radiographic findings that should be considered (11). Although, 2-D images have their limitations, conventional dental radiography is still the most preferred clinical method to assess the long-lasting success of an implant (11,12). Presently, the dental literature is unequivocal on the association of marginal bones loss (MBL) with the implant retention mechanism. Sailer *et al.* (3) stated that cement-retained restorations exhibit more MBL (>2mm) than the screw retained restorations. In contrast, Brandao *et al.* (12) reported that there is limited evidence to reveal differences in the MBL between screw and cement-retained restorations. Another review by Sheriff *et al.* (13) showed that there is no statistically significant difference for major and minor outcomes between screw and cement-retained restorations with regard to the survival of implant or crown loss.

As there is no agreement regarding the superior retention scheme for the implant-supported fixed restorations therefore, a study was planned to compare MBL around implant supported cement-retained versus screw-retained crowns or fixed partial denture prosthesis and to determine various predictors of the MBL

## Material and Methods

A retrospective charts review was carried out at the dental clinics, Aga Khan University Hospital (AKUH) Karachi, Pakistan between February 2017 and June 2017. Exemption from the institutional ethics review committee was taken (ERC No. 4410-Sur-ERC-16) prior to the data collection. Convenience sampling technique was utilized. Sample size was calculated by a calculator (sample size determination in health studies, WHO). The study by Nissan *et al.* (14) was used as a reference. They reported that the mean MBL of the screw-retained restorations was 1.4 ± 0.6 mm whereas for cement-retained restorations, it was found to be 0.69 ± 0.5 mm. Keeping these values as a reference, power of study (1-β) at 95% and level of significance (α) 5%, the required sample size turned out to be 52 implants, since we had two groups, we required 104 implants in the study.

We included all patients who have received at least one dental implant supported fixed prosthesis for at least 12 months. All implants included in the study were root form implants (Zimmer Biomet, Carlsbad, CA, USA)

Patients whose data were incomplete, or were lost to follow-up, history of osteoporosis, metabolic bone diseases, receiving bisphosphonates or radiation therapy and patients who received hybrid prosthesis/ implant over dentures were excluded.

**Figure 1:**
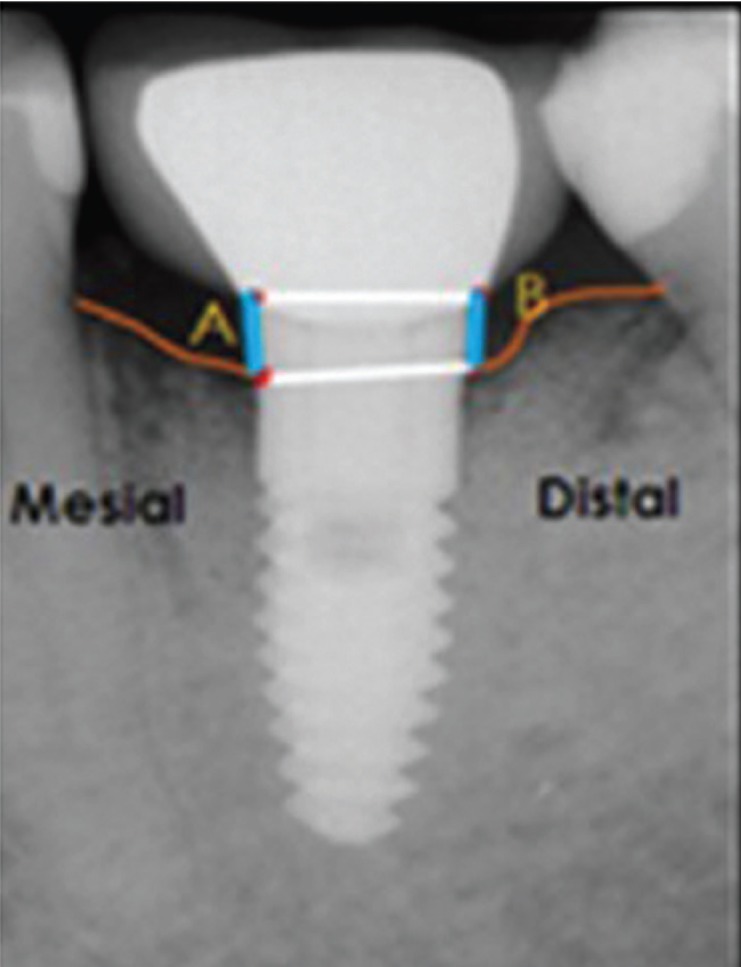
Periapical radiograph and the method employed to calculate the marginal bone loss.

Data was collected on the standardized periapical radiographs that were obtained from the hospital image archives database using SIDEXIS 4 software (Dentsply Sirona, PA, USA) which contains all radiographic scans of the patients visiting the institution. These radiographs were taken during the original treatment planning, execution and at follow-up appointments of the subjects. All radiographs were observed under same conditions on a 15- inch, 1280×800 resolution, 32 bit color mode computer monitor (Dell Inc, TX, USA). The radiographs were assessed by two examiners, who observed all the images simultaneously at one time. The bone height around the implants was measured using the “measuring scale” in the SIDEXIS-4 software. The most coronal part of the implant platform was selected as the reference line from which two perpendicular lines were dropped at the mesial and distal aspect of the implants to the point where first bone to implant contact occurred (Fig. 1). The distance between the most coronal parts to the implant bone interface was calculated. Single reading was made for each proximal site and was used to calculate the amount of crestal bone loss. Subtracting the bone level at 0 month from the bone level at 12 months measured the amount of marginal bone loss (MBL) for both the cement-retained and screw-retained prosthesis. All measurements were recorded on the proforma.

-Data analysis

Individual implant was taken as the unit of analysis. SPSS version 21.0 was used for data analysis. Descriptive statistics including mean and standard deviation was computed for MBL in both the groups. General estimation equation analysis was done to determine the predictors of MBL. A *p*-value < 0.05 was taken as statistically significant.

## Results

One hundred and four implants in 41 patients (21 male patients and 20 female patients); mean age 59.8 ± 13 years, range: 18-84 years) were evaluated. Characteristics of patients are summarized in  Table 1. Distribution of implants with respect to prosthesis, location and diameter is shown in  Table 2. Mean bone loss with respect to type of prosthesis is shown in  Table 3.

**Table 1:**
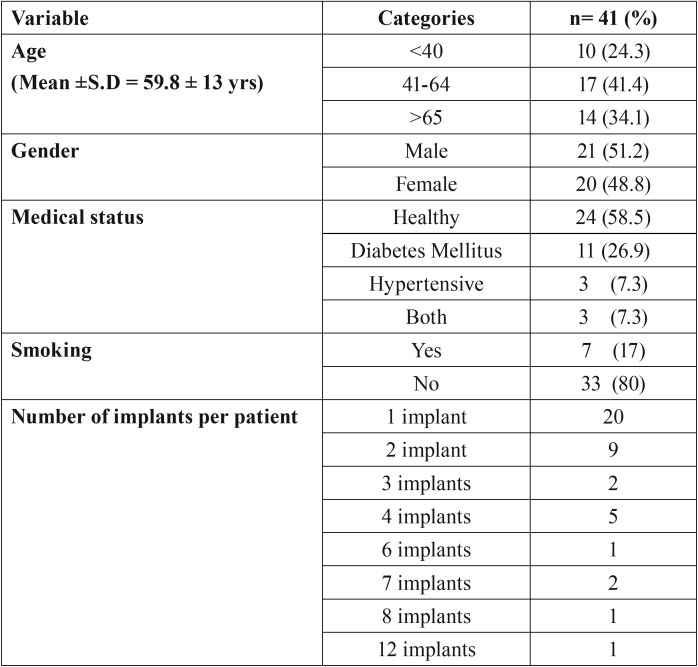
Patient characteristics (n=41).

**Table 2:**
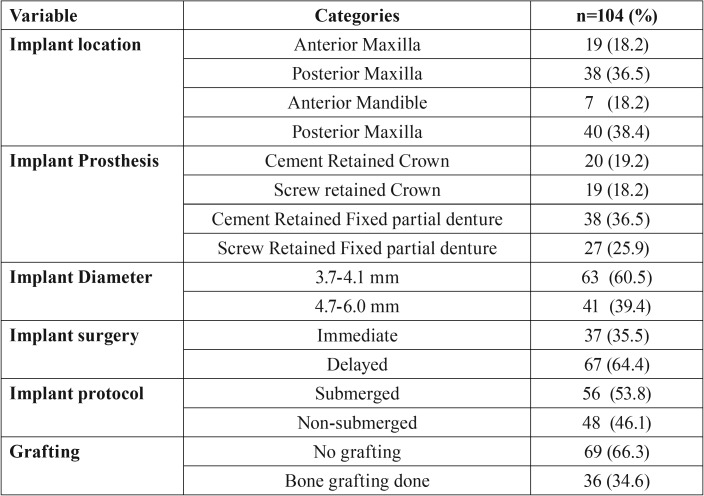
Implant level characteristics (n=104).

**Table 3:**
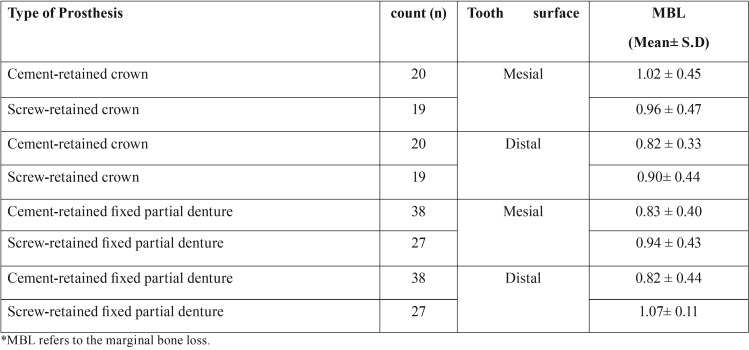
MBL in cement and screw-retained & fixed partial dentures.

Statistically significant differences in MBL were observed b/w screw and cement retained implant prosthesis and screw retained prosthesis had more MBL than cement retained prosthesis (*p*-value =0 .018) (irrespective of crowns or fixed partial dentures) 

Other factors that turned out to be significant predictors of MBL were male gender (*p*-value= <0.01), age >65 yrs. (*p*-value=0.028) and sites where bone grafting was performed (*p*-value=0.003) ( Table 4).

**Table 4:**
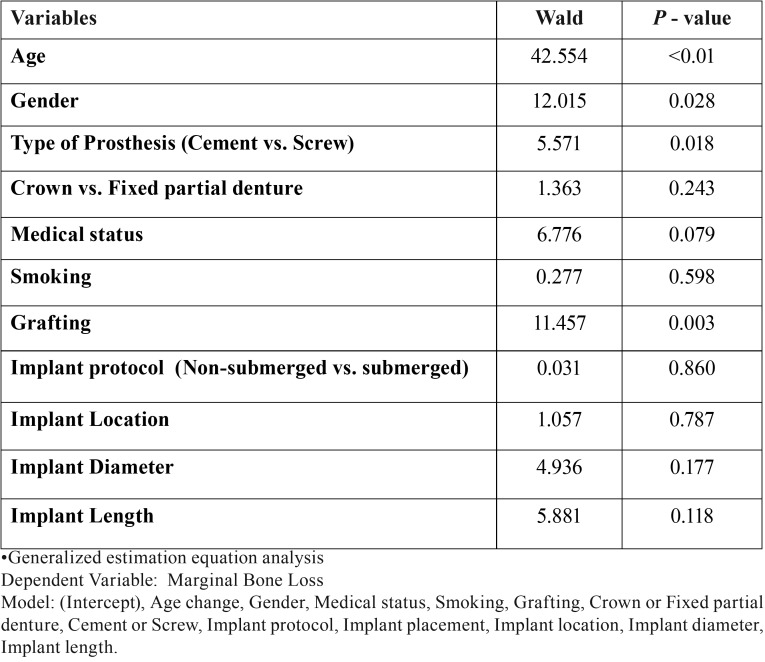
Predictors of Marginal bone loss.

## Discussion

The current study was aimed to determine the marginal bone loss between screw and cement-retained implant prosthesis and the factors that predict MBL.

The general requirement of screw-retained restorations is a precise placement of implant for an optimal location of the screw access needed at the prosthetic phase. Deviation from the optimum position and angulation results in an unaesthetic restoration (15,16). Ceramic may chip off near the access opening in the crown as this opening weakens the integrity of the porcelain (17). However, there are limitations associated with cement-retained restorations too. These present difficulty with the removal of excess cement and pose challenge in gaining access in home based oral hygiene measures (4,18). The problem becomes significant when crown margins of cemented restorations are placed considerably sub-gingival for esthetic reasons, increasing the risk of peri-implantitis (19). Studies consider MBL as an important risk indicator of peri-implantitis (20,21).

In the present study, statistically significant difference was observed when cement-retained prosthesis were compared with the screw-retained prosthesis (*p*-value=0.018) as depicted in  Table 4. This finding was in agreement with the studies conducted by Lemos *et al.* (4) and Nissan et al. (14) but in contrast to the findings reported by Koller *et al.* (19) and Sailer *et al.* (3). A possible explanation for observing greater MBL in screw-retained design could be the fact that the position of the access opening in the prosthetic restoration transfers occlusal loads in a non- axial manner which results in increased marginal bone loss. Studies have shown that there is minimal stress exertion on implant and crestal bone with cement-retained prostheses than with screw-retained prostheses (22-24). Furthermore, literature also suggests that marginal discrepancies at crown margins could be better filled by cements because they can absorb the stresses generated at implant-abutment interface and can compensate for the uneven distribution of the occlusal load (4). In addition to that, reasons such as small number of observations, retrospective study design, non-random group allocation, inability to account for factors such as high masticatory force, occlusion and para-functional habits could also be responsible for having differentially high MBL with screw-retained prosthesis.

What actually constitute a significant MBL is debatable and varies among studies. Koller *et al.* (19) and Adell *et al.* (25) showed that the MBL for implants was 1.5 mm for the first year while Cox and Zarb (26) reported 1.6 mm loss in the first and 0.13 mm in the subsequent years. Thus, having an MBL of 1.5mm is substantially supported by the scientific evidence.

The general estimation equation analysis in the present study was done to identify factors that could probabilistically predict MBL. We observed that advanced age (>65 years), male gender, type of prosthesis and bone grafting are risk indicators of MBL. Similar findings were also documented by Mumcu *et al.* (27) and G-Moreno *et al.* (28) However, there are contrasting observations as well (29,30).

The probable explanation of increased MBL could be attributed to the fact that bone mass density decreases with natural aging. The age-related bone loss predominantly occurs in the cancellous compartment. Increased oxidative stress directly enhances the osteoclastic activity in the trabecular bone and has a very limited effect on the cortical bone (18). In the present study male gender was more affected than females which is contrary to the findings of many studies. The most probable reasons for this could be poor hygiene maintenance, negligence towards brushing and parafunctional habits.

The present study also reveals that MBL is more pronounced in sites that are subjected to bone grafting, these findings are in agreement with study conducted by G-Moreno *et al.* (28) who concluded that there is more marginal bone loss around implants that are placed in augmented bone than implants placed in native bone. Inglam *et al.* (31) in his study reported that the grafted bone is less stiff than that of the native bone, therefore it promotes MBL in grafted sites. Therefore, grafted sites should preferably have stiffness comparable or greater than native bone in order to efficiently deal with the loading and masticatory forces.

The potential limitations to this investigation were small number of observations, retrospective study design, convenience sampling technique, non-random group allocation, inability to account for important confounding factors such as high masticatory force, occlusion, status of opposing dentition and para-functional habits etc.

## Conclusions

Within the limitations of this study, it can be concluded that male patients >65 yrs. with sites needing bone grafts who were provided with screw retained prosthesis (irrespective of crown or fixed partial dentures) had significantly greater marginal bone loss around implants.
